# Achievement emotions in kindergarten: the association of solution accuracy with discrete joy, sadness, and surprise

**DOI:** 10.3389/fpsyg.2024.1466345

**Published:** 2025-01-13

**Authors:** Traci Shizu Kutaka, Pavel Chernyavskiy, Tara Hofkens

**Affiliations:** ^1^School of Education and Human Development, Center for the Advanced Study of Teaching and Learning, University of Virginia, Charlottesville, VA, United States; ^2^Department of Public Health Sciences, University of Virginia, School of Medicine, Charlottesville, VA, United States

**Keywords:** achievement emotions, early childhood mathematics, arithmetic, story problems, automatic facial coding

## Abstract

Children experience a variety of emotions in achievement settings. Yet, mathematics-related emotions other than anxiety are understudied, especially for young children entering primary school. The current study reports the prevalence and intensity of six basic, discrete achievement emotions (joy/happiness, sadness, surprise, anger, fear, and disgust) expressed on the faces of 15 kindergarten-aged children as they solved increasingly complex arithmetic story problems in a 3-month teaching experiment. We also examine how the extent to which the expressed emotions influenced arithmetic accuracy at the end of an instructional session at the beginning, middle, and end of the teaching experiment. Through the application of *FaceReader9*, the three most intensely expressed emotions at the launch of the instructional sessions were happiness/joy, sadness, and surprise. Using functional regressions, these expressed achievement emotions predicted arithmetic accuracy at the end of the instructional session. However, when the effect of session over time was added to the model, the relationship between happiness/joy and accuracy, as well as sadness and accuracy, became non-significant. In contrast, the relationship between surprise and accuracy remained significant. We discuss potential explanations for these patterns of significance and non-significance. This study serves as a critical first step in clarifying how emotions contribute to problem-solving behavior as we grapple with how to respond to the sometimes intense, but always present emotions of young learners in ways that are affirming, as well as mathematically productive and generative.

## 1 Introduction

Modeling and representing the actors and actions that compose a problem-solving situation is a fundamental and strategically challenging mathematical skill (Carpenter et al., 1999), as well as an emotionally-laden process (Muis et al., 2015). The present study focuses on the emotions children experience as they solve mathematical problems. Specifically, we examine patterns of prevalence and intensity of achievement emotions that emerge as children generate solutions to arithmetic story problems and how these achievement emotions are associated with short-term learning outcomes.

Emotions are a coordinated, dynamic subsystem of expressive, affective, cognitive, motivational, and physiological processes (Shuman and Scherer, 2014), with achievement emotions – the central interest of this study – directly tethered to achievement settings (e.g., school), activities (studying for an exam), or outcomes (success or failure on exam) (Pekrun and Stephens, 2010). In Pekrun’s view (2006), achievement emotions can be classified through a three-dimensional taxonomy, organized by valence, activation, and object focus. They are further delineated by positive/pleasant or negative/unpleasant valence and can have a physiologically activating or deactivating effect.

### 1.1 Achievement emotions and mathematics outcomes

McLeod’s (1992) seminal paper on affect (beliefs, attitudes, and emotions) in mathematics education summarizes how emotions have (or have not) been factored into cognitive theories of teaching and learning, noting that “emotional reactions” to mathematics have not received much attention. Studies that have been conducted focused on secondary- and post-secondary learners’ expressions of joy and frustration in solving non-routine problems. McLeod attributes this dearth of studies to the absence of a theoretical framework that clarifies the role of emotions in the learning of mathematics. We second this assertion and add that the methodological complexity of assessing emotions in young children within learning contexts/settings also contributes.

Pekrun and Stephens (2012) have since summarized important advancements in the role of emotions in learning. A large body of research validates the control-value theory of achievement emotions, a framework that organizes the antecedents and consequences of emotions experienced within achievement settings (see: Pekrun, 2006; Pekrun and Linnenbrink-Garcia, 2014; Pekrun and Stephens, 2010), with few studies focusing on mathematics.

Yet, to date, most research has focused on outcome emotions or the consequences of negative activating emotions. Studies on emotions following success or failure are summarized in Weiner (1985, 2007), with mathematics anxiety serving as a prominent example (Beilock et al., 2010; Maloney and Beilock, 2012). However, empirical findings on positive, activating emotions – specifically, enjoyment, hope, pride, and relief – remain rare (Pekrun and Linnenbrink-Garcia, 2014; Pekrun and Stephens, 2012).

### 1.2 The current study

In the current study, we address the following research question: What is the nature (strength, direction, and timing) of the association between discrete achievement emotions expressed by young children as they solve arithmetic story problems and solution accuracy?

### 1.3 Addressing the limitations of past research on achievement emotions

Achievement emotions are typically measured through self-report protocols and questionnaires in primary-aged child and adult samples, which have been found to be psychometrically robust. Yet, scholars also argue that these instruments fail to distinguish one emotion from another (Nicholls, 1976), especially when we rely on conventional survey and interview methods. Zeidner (1995) specifically argues that we may be conflating anxiety with other achievement emotions, such as shame or guilt. Surveys and interviews are also retrospective, and thus fail to capture emotions as they unfold during learning tasks (Hofkens and Ruzek, 2019). Moreover, studies linking emotion to achievement are limited to grades (reported on a Likert scale, as is the practice in Europe, where many of these studies were conducted), as well as standardized test scores (e.g., Dettmers et al., 2011; Wondimu et al., 2013).

The purpose of the current study is to clarify the relationship between achievement emotions and solution accuracy for arithmetic story problems. We expand upon the existing body of research in four ways. First, this study goes beyond anxiety and focuses on state-like academic emotions: basic, universal emotions (Ekman, 1992; Scherer and Ekman, 2014) that are distinct, brief in duration with a quick onset, and therefore follow a fluctuating trajectory over time within an achievement setting or activity. Basic achievement emotions are thus discrete and include joy/happiness, surprise, sadness, anger, disgust, and fear. Second, we move beyond emotions that emerge in response to outcomes (task success or failure) and instead focus on emotions that arise *in situ* during the problem-solving process in a naturalistic setting. Third, we focus on a kindergarten sample – a younger age group than previously studied. Fourth, instead of using questionnaires, self-report instruments, or interview protocols, we analyze moment-to-moment fluctuations in expressed emotion as captured on video documenting the problem-solving process.

## 2 Method

### 2.1 Participants and data collection context

Coded arithmetic accuracy and emotional intensity data come from 15 children (7 girls; aged 5 years) who were enrolled in a semester-long teaching experiment conducted in a Mountain West US state. These children received up to 15 one-on-one instructional sessions. The content of instruction followed a validated arithmetic Learning Trajectory (Sarama, 2009). Further details of the teaching experiment and of its efficacy can be found in Clements et al. (2020).

### 2.2 Video data collection

All instructional sessions were videotaped by a GoPro^©^ camera (Grant # Blinded), coded for accuracy and problem-solving strategy sophistication (Kutaka et al., 2023; Grant # Blinded), and then analyzed in the current study by FaceReader9 software (Noldus FaceReader 9, 2022) to encode the intensity of discrete universal emotions exhibited by each child (Grant # Blinded). For each of the 15 children, we selected a random set of their instructional sessions, such that the beginning (sessions 1–5), middle (6–10), and end (11–15) of their participation in the experiment was represented. Exclusion criteria is specified in the Supplementary material. The final analytic sample reflects 67 coded instructional sessions across 15 participants; at least three sessions per participant, on average 4.7 sessions, and at most nine sessions.

### 2.3 Instruments

#### 2.3.1 FaceReader9 software

This software is built on the work of the Facial Action Coding System (FACS) group to automatically encode six basic, discrete emotions, as well as neutral states. This is accomplished through the software’s analysis of facial muscles, valence, arousal, gaze direction, and head orientation, as well as subject gender and age. For each video frame, FaceReader9 (i) locates the face, (ii) overlays a dynamic mesh over the face to digitally capture facial musculature and gaze, and (iii) uses a neural network to estimate the *degree of concordance* between the displayed facial expression and the database of emotion-specific expressions. This degree of concordance (continuous 0–1 variable) is called *emotional intensity*, where 0 = “not visible”; 0.2 = “slightly visible”; 0.5 = “clearly visible”; and 1 = “prototypical emotion” (van Kuilenburg et al., 2005). The theoretical foundation and accuracy of this tool is described in the Supplementary material.

#### 2.3.2 Arithmetic accuracy

Accuracy was originally coded using six levels: Correct; Correct with Support of Instructor; Incorrect; Incorrect with the Support of Instructor; “I do not know” statement by child; and “No response.” A solution was considered as correct where 1 = Correct if their response was “Correct” or “Correct with Support of Instructor” and “Incorrect” otherwise. To compute session-level accuracy, operationalized as the proportion of correct solutions, we averaged solution correctness code over all attempts per instructional session to generate a percent correct for each child.

### 2.4 Statistical analysis

Emotional intensity – defined as a continuous metric ranging between 0 and 1 for each universal discrete emotion – was encoded by FR per video frame and averaged within one second of video for analysis. We analyzed emotional intensity throughout the session/task using Functional data analysis (FDA) techniques, as has been done in the context of emotion dynamics (Kuppens et al., 2009; Kuppens and Verduyn, 2017). FDA describes a family of analytic techniques developed for explanatory and response variables that comprise a function or curve (Ramsay et al., 2009). We used emotional intensity curves coded by FR as functional explanatory variables in our analysis.

The functional variables were constructed using intensity for 40 to 250 s (210 s) of instruction within each instructional session. We analyzed this sub-section of the session for two reasons. First, the launch phase of an inquiry-oriented math task is fundamental for engaging children in effective authentic problem-solving: it orients the child to the task and activates the prior knowledge they will use to interpret and solve the problem (Van de Walle et al., 2023). Second, this timeframe enables us to focus on the achievement emotions evoked during the first problem-solving event so we can observe its cascading effects on session-level learning and performance.

An R markdown file detailing the analysis is provided at http://github.com/pchernya/Achieve_EMO_Frontiers to facilitate reproducibility. Deidentified data sharing can take place following reasonable request under existing data sharing agreements.

A functional regression was estimated using the refund (Goldsmith et al., 2023) R package, analyzing one emotion at-a-time. We interpolated portions of emotional intensity when children momentarily turned away from the camera: an average of 13.4% of the video frames were interpolated via a non-parametric kernel regression with an adaptive nearest neighbor algorithm in the np R package (Hayfield and Racine, 2008). The resultant emotional intensity functions for the three most intense emotions (joy/happiness, sadness, surprise) were used to predict average session accuracy. We additionally include a student random intercept and a session effect. We used the likelihood ratio test for all hypothesis testing; *p*-values<0.05 were considered “statistically significant”.

## 3 Results

When children did not display a neutral facial expression, the three most intensely displayed discrete emotions were happiness/joy, sadness, and surprise over the initial 210 s of the instructional session. We therefore focus on the contributions of these three emotional states, operationalized as functional predictors of session-level arithmetic accuracy for story problems.

The baseline model only included a student random intercept. The model that directly tests the hypothesis that emotions were an important contributor to session-level accuracy adds the emotional intensity functional predictor to the baseline model. Finally, to test whether the effect of emotions persists after accounting for the effect of instructional session (i.e., time spent in the teaching experiment), we additionally include the linear session effect. The addition of child gender or the instructor random intercept were not statistically significant and did not alter any of the interpretations of the emotional functional predictors and were omitted.

**Figure 1:**
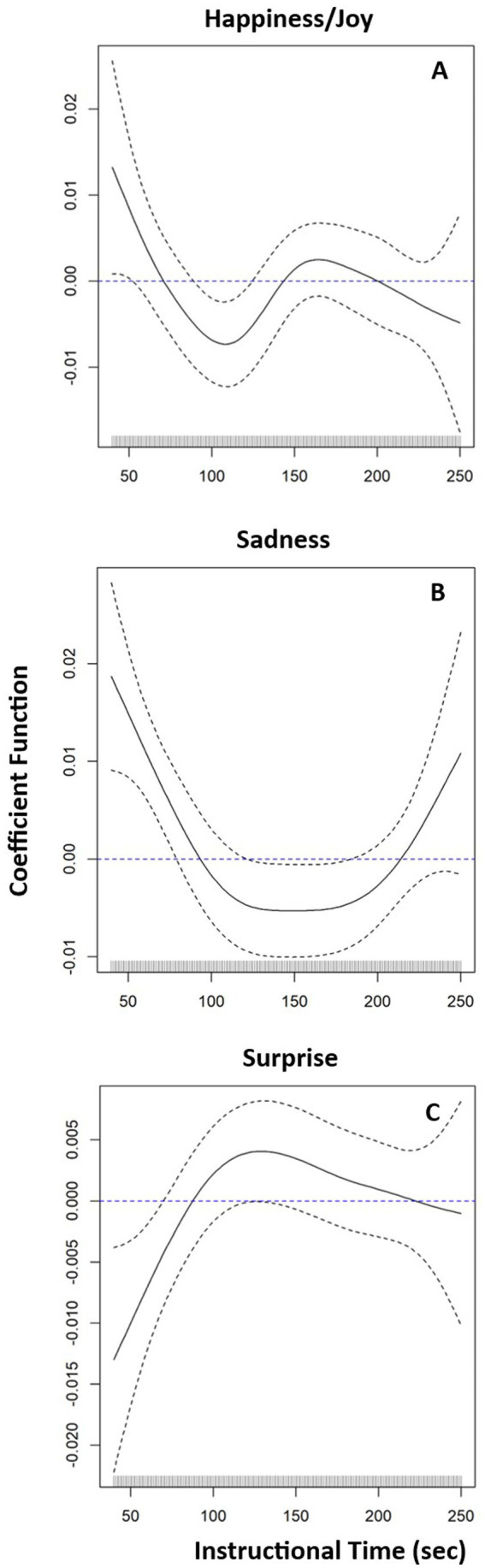
Time-varying effects (solid lines) of discrete happiness/joy **(A)**, sadness **(B)**, and surprise **(C)** on session level solution accuracy. The dotted lines represent the 95% Confidence Intervals.

The baseline model explained 55.8% of the variability in session-level arithmetic accuracy. The addition of happiness/joy, as a functional predictor, was statistically significant (*χ*^2 (4.16) = 14.70, *p* = 0.01) and explained an additional 9.3% of variability in session-level arithmetic accuracy. Happiness/joy had a time-varying effect (Figure 1A): more intense initial happiness/joy predicted higher accuracy. However, this positive effect on accuracy reversed and became negative over approximately the next 120 s of instruction, then null after 150 s of instruction. After adding the effect of session, the relationship between happiness/joy and accuracy was non-significant *χ*^2 (4.41) = 4.41, *p* = 0.06.

The addition of sadness as a functional predictor to the baseline model was also statistically significant (*χ*^2 (4.90) = 29.31, *p* < 0.01), explaining an additional 16.7% of variability in accuracy. More intense initial sadness predicted higher accuracy for the first 100 s of instruction, lower accuracy from 100–200 s of instruction, becoming null thereafter (Figure 1B). After adding the effect of session, the relationship between sadness and accuracy remained significant (χ^2 (5.63) = 17.65, *p* = 0.01). Together with the session effect, this model explained 73.9% of the variation in accuracy.

Likewise, the addition of surprise was statistically significant (χ^2 (4.01) = 11.65, *p* = 0.02), explaining an additional 7.5% of variability over the baseline model. More intense surprise predicts lower accuracy until 100 s of instruction, becoming null thereafter (Figure 1C). After adding the effect of session, the relationship between surprise and accuracy remained significant (χ^2 (4.11) = 12.44, *p* = 0.01). Together with the session effect, this model explained 69.6% of the variation in accuracy.

## 4 Discussion

This brief report serves as proof-of-concept that validated and theoretically grounded automatic facial coding software can clarify how achievement emotion intensity is associated with short-term learning outcomes for young children. Discrete, universal achievement emotions in the first 240 s predicted arithmetic learning based on performance across an entire instructional session. Indeed, the implementation of FACS coding in FaceReader addresses two major limitations in the literature describing the achievement emotions of young children. First, this tool enables the field to move beyond reliance on ad-hoc self-reporting measures; we can instead access children’s expressed emotions as they work through the problem-solving process from one moment to the next. Second, this type of facial coding and analysis can be applied at scale. The contributions shared here are meant to be hypothesis-generating. Below we offer potential explanations for why discrete expressions of joy/happiness, sadness, and surprise may impact short-term learning, but further qualitative and mixed methods work is needed to substantiate our preliminary findings.

Initial expressions of joy/happiness positively contribute to solution accuracy. This is consistent with the control-value theory of achievement emotions, which accounts for the association between positive, activating emotions and achievement (Pekrun, 2006; Pekrun et al., 2009; Pekrun and Stephens, 2010). The Broaden and Build theory additionally offers a mechanistic explanation for this relationship. Stifter et al. (2020) summarizes the research on the experimental evidence for the broadening function of positive emotions for academic outcomes in young children, reporting that when positive affect was elicited, cognitive performance in preschool improved (Blau and Klein, 2010). Similarly, first- and second-grade children who exhibited positive affective states demonstrated greater visuo-spatial reasoning and organization, as well as cognitive flexibility relative to conditions that elicited negative or neutral affect (Rader and Hughes, 2005). In this study, the effect of happiness/joy is attenuated when we include the effect of session (i.e., time) within the teaching experiment. One possible explanation for this waning association is that accuracy became expected as competencies solidified, and a correct solution was no longer a particularly joyous moment.

Considering the research on the detrimental impacts of negative emotions (anxiety, frustration), we found the positive association between initial discrete sadness and accuracy counterintuitive. However, there is research that challenges the assumption that deactivating emotions such as sadness are negatively associated with exploration and learning behaviors (Bakic et al., 2015). In particular, Di Leo et al. (2019) examined the sequence of achievement and epistemic emotions elementary-aged students experience, where emotions functioned as mediators between task value/control and problem-solving performance. Students reported high rates of negative emotions during a complex problem-solving task with multiple entry points. Negative emotions – frustration, confusion, and boredom – precited planning, cognitive strategies, and meta-cognitive learning strategies, which in turn, predicted task performance. There may be comparable patterns or sequences of emotions and problem-solving behavior among kindergarten students. Another possible interpretation regards the resiliency of young children in this achievement setting. Indeed, instructors are posing story problems explicitly designed to push the thinking of individual children forward at that particular moment in their arithmetic development. Yet, despite expressing a facial configuration that indicates some level of sadness, their accuracy is not compromised. Importantly, consistent with both interpretations, our results suggest that sustained expressions of sadness (i.e., longer than 100 s) had the expected adverse effect on accuracy.

Surprise is a neutral epistemic emotion (Chevrier et al., 2019) whose function has not received a lot of attention in the context of early mathematical learning and development (beyond approximate number in infant studies of cognition). Some research on this emotion suggests it is a “powerful force that can shape what and when children learn” (Stahl and Feigenson, 2019, p. 137), with the stipulation that perceived incongruities and contradictions are resolved and confusion does not linger (D’Mello and Graesser, 2012). We found that initial expressions of surprise contribute to lower levels of accuracy. A review by Munnich and Ranney (2019) offers a potential explanation: low levels of surprise are associated with learning and achievement, such that low surprise allows for the assimilation of destabilizing information into mental representations and concepts (see also: Maguire et al., 2011). This explanation is built on a two-stage model of surprise, such that initial surprise (a violation of expectation) is followed by resultant surprise, where intensity varies as a function of an individual’s ability to explain or make sense of this initial violation. Similarly, Di Leo et al. (2019) found that surprise initiates two potential subsequent emotions depending on the learner’s appraisal of how difficult it would be to solve the problem: curiosity or confusion. Unlike other research with adults, where confusion can result in learners rallying effective strategies and resources (D’Mello et al., 2014; Silvia, 2010), surprise transitioned to curiosity, confusion, and/or frustration, which they hypothesize may undermine accuracy by potentially overwhelming young learners. In other words, children might not yet have the skills needed to respond to this state in mathematically productive and generative ways. This may also account for why other studies suggest that predictability (in contrast to violations of expectations) is advantageous for learning (Benitez and Saffran, 2018).

## 5 Conclusion

Our findings serve as a reminder that learning does not require constant joy/happiness or the absence of all negative emotion (in the short term). Learning and development are not easy processes to embody – and this is authentically and sincerely expressed on the faces of young children when they encounter difficult problems that are explicitly designed for their mathematical benefit. It bears asking, then, what the content of instruction should contain when a child is expressing intense emotions in the process of solving a story problem. Relatedly, how can teachers respond to the emotions of young learners in ways that are affirming, as well as mathematically productive and generative? Indeed, the apogee of this research is to construct emotionally-responsive teaching practices, routines, and interventions that support early problem-solving outcomes. To do so, a critical first step is to characterize how mathematical content interacts with achievement and epistemic emotions in ways that constrain or facilitate learning.

### 5.1 Future directions

Despite addressing several gaps in the current literature on achievement emotions, capturing and analyzing real-time emotions is a complex endeavor; consequently, we acknowledge five limitations. First, due to the strict exclusion criteria of instructional videos (see Supplementary material), our sample size was modest and further research should validate the robustness of these findings through larger, representative samples. Second, we make inferences about the prevalence and intensity of emotional expressions generated from facial coding software. What was encoded may or may not align with self-report measures. Although we follow Martinez’s (2019) recommendations for best practices for research employing this software, as well as Barrett et al.'s (2019) terminology, mixed and qualitative methods can investigate concurrence and contradiction in alignment between lived experience and what is encoded. Third, other achievement and epistemic emotions were likely present (e.g., boredom; curiosity), but not measured by FaceReader9. Fourth, we report discrete emotional states, but blended emotions (e.g., happiness + sadness = melancholy) might be expressed in a sample this young (Denham, 2023). Finally, although we focus on accuracy here, investigating other short-term learning outcomes may be worthwhile, including problem-solving strategy sophistication (Kutaka et al., 2023).

Beyond the limitations of the present study, we also see several avenues for extending this work. The current study speaks to the contributions or consequences of emotions, but does not address the antecedents, as other studies have (e.g., Di Leo et al., 2019). A novel contribution to the antecedent research is to go beyond control-value theory components and focus instead on features of the task, the content of instruction, and their interaction. This includes investigating whether emotions mediate the relationship between features of the task and learning outcomes (Pekrun, 2006).

## Data Availability

The data analyzed in this study is subject to the following licenses/restrictions: original data under embargo at the time of writing. Requests to access these datasets should be directed to traci.kutaka@virginia.edu.
