# Supplementary material for: Achievement emotions in kindergarten: the association of solution accuracy with discrete joy, sadness, and surprise
Source: Front Psychol. 2025 Jan 13;15:1466345. doi: 10.3389/fpsyg.2024.1466345 (PMC11770055; doi:10.3389/fpsyg.2024.1466345)
Supplement: Supplementary file 1 [file Table_1.DOCX]

Supplementary Material

# Supplementary Methods

**Theoretical Foundation of *FaceReader09***

The theoretical foundation of *FaceReader9* software (FR) is built upon the well-established Facial Action Coding System (Ekman & Friesen, 1978; Ekman & Rosenberg, 2005) (FACS), where the movement of 44 facial muscles is scored on a 5-point scale and translated into an “intensity” score. Because all humans have nearly identical facial muscle anatomy (Schmidt & Cohn, 2001), FACS – and therefore FR – scoring for universal discrete emotions is widely generalizable and comparable between individuals regardless of demographics.

***FaceReader* Accuracy.** Implementation of FACS coding in FR is accurate overall (88%), slightly better in women (89%) than men (86%) (Lewinski et al., 2014). Accuracy for coding *joy/happiness*, *anger*, *surprise*, and *sadness* tends to be higher than the accuracy for *fear* and *disgust* (Höfling et al., 2022; Küntzler et al., 2021). Older versions of FR have demonstrated satisfactory accuracy on large and diverse databases of facial expressions (Lewinski, 2015; Skiendziel et al., 2019), as well as near perfect test-retest reliability (Borsos et al., 2022) and robustness to viewpoint angles up to 30 degrees (Namba et al., 2021).

**Video Exclusion Criteria**

Because the video recordings were not originally intended for facial coding, camera placement varied by instructor, physical location of the instructional session, and day of instruction. Instructors placed the camera to capture the interaction of the child with the manipulatives provided for the session; however, the child’s face was nearly always in the frame.

Videos of instructional sessions were excluded prior to encoding by *FaceReader9* if any of the following occurred: 1) the instructor’s face was in the frame next to the child; 2) the participating child’s face was not clearly visible due to camera placement or lighting conditions; 3) the instructional session was disrupted, such that the child was out of the frame for an extended period of time; or 4) there were multiple children in the frame at any point during the video. After coding by *FaceReader9*, instructional sessions were excluded if any of the following occurred: 1) the software reported that the facial model quality was “poor”; or 2) more than 30% of the frames could not be coded for any reason.

**Establishing Inter-Rater Agreement**

For this study, inter-rater agreement is expressed as a percentage (Gisev et al., 2013). The coding team consisted of three graduate students and the Co-PI. Team members became eligible to code independently when they achieved an inter-rater agreement score of 90% against the master coder, for two instructional sessions. In other words, an individual needed to endorse an identical code to the master coder for at least 90% of the problem-solving events within an instructional session. Once team members began coding independently, they continued to meet in pairs to establish agreement for videos that were double-coded.

To check the coders’ degree of agreement over time and prevent coder drift, weekly meetings were held with the Co-PI to ask general questions, and amongst coding pairs to ensure high inter-rater agreement (Bao et al., 2009; Jensen et al., 2010). Across six pairs of coding teams, inter-rater agreement ranged from 85% to 99%, with an overall agreement score of 86%.

**References**

Bao, S., Howard, N., Spielholz, P., Silverstein, B., & Polissar, N. (2009). Interrater reliability of posture observations. *Human Factors*, *51*(3), 292–309.

Borsos, Z., Jakab, Z., Stefanik, K., Bogdán, B., & Gyori, M. (2022). Test–Retest Reliability in Automated Emotional Facial Expression Analysis: Exploring FaceReader 8.0 on Data from Typically Developing Children and Children with Autism. *Applied Sciences*, *12*(15), 7759. https://doi.org/10.3390/app12157759

Ekman, P., & Friesen, W. V. (1978). Facial Action Coding System [dataset]. In *Environmental Psychology \& Nonverbal Behavior*. https://doi.org/10.1037/t27734-000

Ekman, P., & Rosenberg, E. L. (2005). *What the Face RevealsBasic and Applied Studies of Spontaneous Expression Using the Facial Action Coding System (FACS)*. Oxford University Press. https://doi.org/10.1093/acprof:oso/9780195179644.001.0001

Gisev, N., Bell, J. S., & Chen, T. F. (2013). Interrater agreement and interrater reliability: Key concepts, approaches, and applications. *Research in Social and Administrative Pharmacy*, *9*(3), 330–338.

Höfling, T. T. A., Alpers, G. W., Büdenbender, B., Föhl, U., & Gerdes, A. B. M. (2022). What’s in a face: Automatic facial coding of untrained study participants compared to standardized inventories. *PLOS ONE*, *17*(3), e0263863. https://doi.org/10.1371/journal.pone.0263863

Jensen, B. F., Gulbrandsen, P., Benth, J. S., Dahl, F. A., Krupat, E., & Finset, A. (2010). Interrater reliability for the Four Habits Coding Scheme as part of a randomized controlled trial. *Patient Education and Counseling*, *80*(3), 405–409.

Küntzler, T., Höfling, T. T. A., & Alpers, G. W. (2021). Automatic Facial Expression Recognition in Standardized and Non-standardized Emotional Expressions. *Frontiers in Psychology*, *12*, 627561. https://doi.org/10.3389/fpsyg.2021.627561

Lewinski, P. (2015). Automated facial coding software outperforms people in recognizing neutral faces as neutral from standardized datasets. *Frontiers in Psychology*, *6*. https://doi.org/10.3389/fpsyg.2015.01386

Lewinski, P., den Uyl, T. M., & Butler, C. (2014). Automated facial coding: Validation of basic emotions and FACS AUs in FaceReader. *Journal of Neuroscience, Psychology, and Economics*, *7*(4), 227–236. https://doi.org/10.1037/npe0000028

Namba, S., Sato, W., & Yoshikawa, S. (2021). Viewpoint Robustness of Automated Facial Action Unit Detection Systems. *Applied Sciences*, *11*(23), 11171. https://doi.org/10.3390/app112311171

Schmidt, K. L., & Cohn, J. F. (2001). Human facial expressions as adaptations: Evolutionary questions in facial expression research. *American Journal of Physical Anthropology*, *116*(S33), 3–24. https://doi.org/10.1002/ajpa.20001

Skiendziel, T., Rösch, A. G., & Schultheiss, O. C. (2019). Assessing the convergent validity between the automated emotion recognition software Noldus FaceReader 7 and Facial Action Coding System Scoring. *PLOS ONE*, *14*(10), e0223905. https://doi.org/10.1371/journal.pone.0223905
